# Genetic Landscape of Lynch Syndrome in a High-Risk Serbian Cohort: Predominance of *MLH1* Variants and Implications for Risk-Based Testing

**DOI:** 10.3390/ijms27156652

**Published:** 2026-07-25

**Authors:** Marija Djordjic Crnogorac, Valentina Karadzic, Teodora Cato, Milena Cavic, Neda Nikolic, Jelena Spasic, Fedja Djordjevic, Vladimir Jokic, Milan Kocic, Miroslav Djurasinovic, Biljana Kukic, Srdjan Nikolic, Marija Ristic, Marijana Milovic, Ana Krivokuca

**Affiliations:** 1 Department for Genetic Counseling for Hereditary Cancer, Institute for Oncology and Radiology of Serbia, Pasterova 14, 11000 Belgrade, Serbia; valentina.karadzic@gmail.com; 2 Department for Experimental Oncology, Institute for Oncology and Radiology of Serbia, Pasterova 14, 11000 Belgrade, Serbia; teodoracato2101@gmail.com (T.C.); milena.cavic@ncrc.ac.rs (M.C.); 3 Clinic for Medical Oncology, Institute for Oncology and Radiology of Serbia, Pasterova 14, 11000 Belgrade, Serbia; nedanikolic@hotmail.com (N.N.); jelena.spasic@ncrc.ac.rs (J.S.); djordjevicfedja@gmail.com (F.D.); miroslav.dj@ncrc.ac.rs (M.D.); moxi.bg@gmail.com (M.R.); marijana.milovic.kovacevic@gmail.com (M.M.); 4 Clinic for Surgical Oncology, Institute for Oncology and Radiology of Serbia, Pasterova 14, 11000 Belgrade, Serbia; vladimir.jokic90@gmail.com (V.J.); kocic011@gmail.com (M.K.); onkosurge1@yahoo.com (S.N.); 5 Department for Medical Oncology, Oncology Institute of Vojvodina, University of Novi Sad, 21000 Novi Sad, Serbia; bikikuki@gmail.com; 6 Faculty of Medicine, University of Belgrade, 11000 Belgrade, Serbia

**Keywords:** developing countries, genetic testing criteria, hereditary nonpolyposis colorectal cancer, Lynch syndrome, *MLH1* mutations, genetic screening

## Abstract

Lynch syndrome (LS) is the most common hereditary colorectal cancer (CRC) syndrome, caused by germline pathogenic or likely pathogenic variants (PV/LPV) in mismatch repair (MMR) genes. Data on the spectrum of LS-associated variants in Slavic populations, including Serbia, remain limited. Given the high burden of CRC and endometrial cancer and the limited implementation of hereditary CRC screening, characterizing the spectrum of germline variants in clinically selected high-risk individuals is important for improving genetic testing strategies, risk assessment, and clinical management. Between 2018 and 2025, 176 individuals underwent germline testing for hereditary CRC syndrome based on the Amsterdam/Bethesda criteria, validated LS risk prediction models, and/or family history (FH). Next-generation sequencing (NGS) was performed using the Illumina TruSight Hereditary Cancer Panel, and variants were classified according to American College of Medical Genetics and Genomics and Association for Molecular Pathology (ACMG/AMP) guidelines. PV/LPVs in MMR genes were identified in 27/176 (15.3%) and were associated with positive FH of LS-related tumors (*p* = 0.0001). Most PV/LPVs were identified in *MLH1* (10.2%), followed by *MSH2* (4.0%) and *MSH6* (1.1%), with no PV/LPVs identified in *PMS2*. Additionally, no pathogenic sequence-level *EPCAM* variants detectable by the applied panel-based NGS approach were identified. Recurrent *MLH1* variants were observed in multiple families, and two previously unreported *MLH1* variants were identified. This first systematic analysis of a clinically selected high-risk Serbian cohort provides novel data on the spectrum of LS-associated variants in this referral population, demonstrates the predominance of *MLH1* variants, and supports broader implementation of genetic testing, tumor screening, and genetic counseling.

## 1. Introduction

Lynch syndrome (LS), which is also known as hereditary nonpolyposis colorectal cancer (HNPCC), is the most frequent hereditary colorectal cancer syndrome accounting for 2–3% of all colorectal cancer (CRC) cases [1]. It is caused by germline pathogenic variants (PV) or likely pathogenic variants (LPV) in the DNA mismatch repair (MMR) genes *MLH1*, *MSH2*, *MSH6*, and *PMS2*, or by specific *EPCAM* deletions associated with *MSH2* silencing. Dysfunction of these genes leads to the accumulation of genetic errors, increasing the probability of cancer development [2,3]. In addition to the risk of CRC, individuals with LS have an increased lifetime risk of endometrial cancer (EC), ovarian cancers ( OC), as well as several other types of extracolonic cancer [3].

The likelihood of developing cancer is influenced by both the affected MMR gene and sex. Carriers of PV/LPVs in the *MLH1* and *MSH2* genes face the highest penetrance, with a lifetime risk of CRC reaching approximately 50% by age 70. In contrast, individuals with *MSH6* and *PMS2* PV/LPVs have a lower, yet still substantial, CRC risk—particularly *MSH6* carriers, who may encounter a lifetime risk of around 30% by age 70. The risk of EC is also significant among carriers of MMR gene PV/LPVs, especially for those with *MLH1*, *MSH2*, and *MSH6* ones, where the risk ranges from 40 to 60% by age 70 [3,4]. Recent research emphasizes the importance of CRC surveillance strategies tailored to specific MMR gene PV/LPVs in LS patients. For those with *MLH1* and *MSH2* PV/LPVs, intensive surveillance should begin at age 25, with screening every 1–2 years. For carriers of *MSH6* and *PMS2* PV/LPVs, it may be more cost-effective to start surveillance later and extend the intervals between screenings [5]. Despite these advances, there is no universally standardized protocol for LS screening. Current guidelines instead emphasize the need to tailor screening strategies to national contexts, reflecting differences in scientific infrastructure, clinical practice, logistics, and healthcare education systems across countries [6].

Recent European studies have improved our understanding of the prevalence of LS. European guidelines from the European Hereditary Tumour Group (EHTG) and the European Society of Coloproctology (ESCP) estimate that the prevalence of pathogenic MMR gene carriers is approximately one in 300 individuals. This translates to about 2.5 million people across Europe [7]. However, data on the prevalence and mutational spectrum of LS in Slavic populations remain limited and fragmented. Although studies have been conducted in individual populations, including Russian, Slovenian, Macedonian, Polish, and Czech cohorts, comprehensive and regionally integrated data are still lacking [8,9,10,11,12].

In Serbia, CRC represents a major public health burden. According to the most recent estimates, it is the second leading cause of cancer-related mortality among men and the third among women, with age-standardized incidence rates of approximately 45–50 per 100,000 in men and 25–30 per 100,000 in women, and mortality rates of around 25–30 and 12–15 per 100,000, respectively [13].

In addition, EC is the fifth most common malignancy among women in Serbia, with an incidence rate of approximately 15–20 per 100,000 and a mortality rate of around 4–6 per 100,000. These data further emphasize the need for improved awareness, early detection, and effective management of hereditary cancer predisposition [13]. These epidemiological patterns are particularly concerning in the context of hereditary cancer syndromes, where early identification of high-risk individuals remains suboptimal.

Together, these incidence and mortality rates underscore the pressing challenges faced in oncology and the importance of research and support for high-risk individuals and their families. Although significant improvements in CRC management in Serbia have been reported in the last 20 years [14,15], systemic solutions will be crucial for tackling both the observed high incidence of young-onset CRC [16,17] and the growing elderly population.

Although Serbia has implemented a national CRC screening program for the average-risk population, no comparable nationwide framework exists for the identification and management of individuals with hereditary CRC syndromes. The absence of an organized, nationwide CRC screening program for the high-risk setting, coupled with the limited implementation of genetic testing for hereditary CRC syndromes, represents a significant gap in cancer prevention and early detection. Consequently, carriers of high-risk pathogenic variants often remain undiagnosed until advanced disease stages. Identifying these individuals systematically would facilitate timely surveillance and targeted interventions, thereby improving patient outcomes and enhancing the cost-effectiveness and long-term sustainability of CRC management at a national level. With all this in mind, there is a clear need for national data from clinically characterized high-risk individuals to inform tailored genetic testing and surveillance strategies.

To address this unmet need, we performed the first systematic analysis of germline MMR gene variants in a clinically selected high-risk Serbian cohort. By characterizing the frequency and spectrum of germline MMR variants in this referral population, the present study provides novel data on LS genetics from an underrepresented region of Europe.

## 2. Results

### 2.1. Study Population

**Figure 1:**
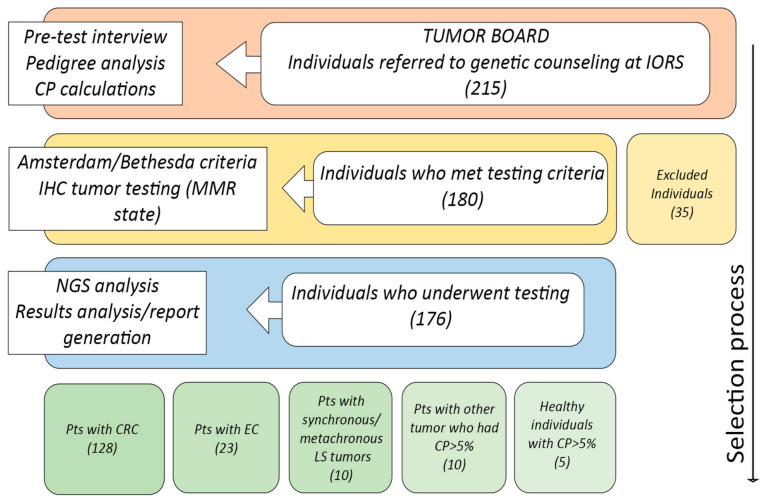
Flowchart illustrating selection of subjects and inclusion in the Lynch syndrome (LS) genetic testing cohort. Abbreviations: Institute for Oncology and Radiology of Serbia (IORS), immunohistochemistry (IHC), mismatch repair (MMR), next-generation sequencing (NGS), patients (Pts), colorectal cancer (CRC), endometrial cancer (EC), Lynch syndrome (LS), predicted probability of LS calculated using validated risk prediction models (CP).

Of the 180 subjects who met the LS testing criteria described in the Section 4, 176 underwent germline testing by October 2025 (Figure 1, Table 1). The majority were patients with CRC (128/176, 72.7%; Group 1), followed by individuals diagnosed with EC (23/176, 13.1%; Group 2). Ten (5.7%) of 176 individuals were patients with multiple primary LS tumors, including CRC, EC, OC, duodenal and/or pancreatic cancer. An additional 10 patients (5.7%; Group 4) were diagnosed with malignancies other than CRC or EC, including both other LS–associated tumors (e.g., pancreatic cancer) and cancers not classically linked to LS, such as breast or Fallopian tube cancer. Finally, 5 individuals (2.8%; Group 5) were unaffected but referred for testing due to a calculated carrier probability >5% and/or a positive FH of first- or second-degree relatives with early-onset LS-associated cancer.

### 2.2. Frequency of Pathogenic/Likely Pathogenic Variants in MMR Genes

Genomic DNA was isolated using standard laboratory techniques at optimal concentrations and analyzed to identify germline variants in MMR genes. In this group of 176 individuals tested for LS, PV/LPVs were found in 27 cases (15.3%) in the *MLH1*, *MSH2*, and *MSH6* genes (Table 2). No PV/LPVs were identified in *PMS2*, and no pathogenic *EPCAM* alterations detectable by the applied panel-based next-generation sequencing (NGS) approach were identified in this cohort. The majority of detected MMR PV/LPV findings involved the *MLH1* gene (18 findings, 10.2%), while 7 (4.0%) and 2 (1.1%) findings were detected in the *MSH2* and *MSH6* genes, respectively. Among the *MLH1* PV/LPVs identified in the cohort, two recurrent PVs were observed. The variants c.392C>G (p.Ser131Ter) and c.1275dupG (p.Gln426AlafsTer4) were each detected in three unrelated families. The c.1275dupG (p.Gln426AlafsTer4) variant was identified in four individuals, including two individuals from the same family, corresponding to three unrelated families. Two previously unreported *MLH1* variants were identified: the frameshift variant c.496_497insCACTT (p.Leu166SerfsTer3), classified as pathogenic, and the in-frame deletion c.1759_1767delATGCTTGCC (p.Met587_Ala589del), classified as likely pathogenic according to the American College of Medical Genetics and Genomics and Association for Molecular Pathology (ACMG/AMP) framework. The c.496_497insCACTT (p.Leu166SerfsTer3) variant was detected in two unrelated young patients with LS-associated malignancies, and loss of MLH1/PMS2 expression was documented in the tumor of one patient. No variant-specific functional or RNA evidence was available. Detailed classification criteria are provided in Supplementary Table S4.

MMR immunohistochemistry (IHC) results were available for 40 tumors. MMR deficiency (dMMR) was observed in 27 cases (67.5%), of which 13 (48.1%) carried a germline PV/LPV in an MMR gene. The remaining 14 dMMR tumors (51.9%) had no identifiable germline MMR PV/LPV. Retained MMR protein expression (proficient MMR, pMMR) was observed in 13 tumors (32.5%). The distribution of MMR IHC patterns and their corresponding germline findings is summarized in Table 3. Detailed individual IHC and germline results are provided in Supplementary Table S3.

**Figure 2:**
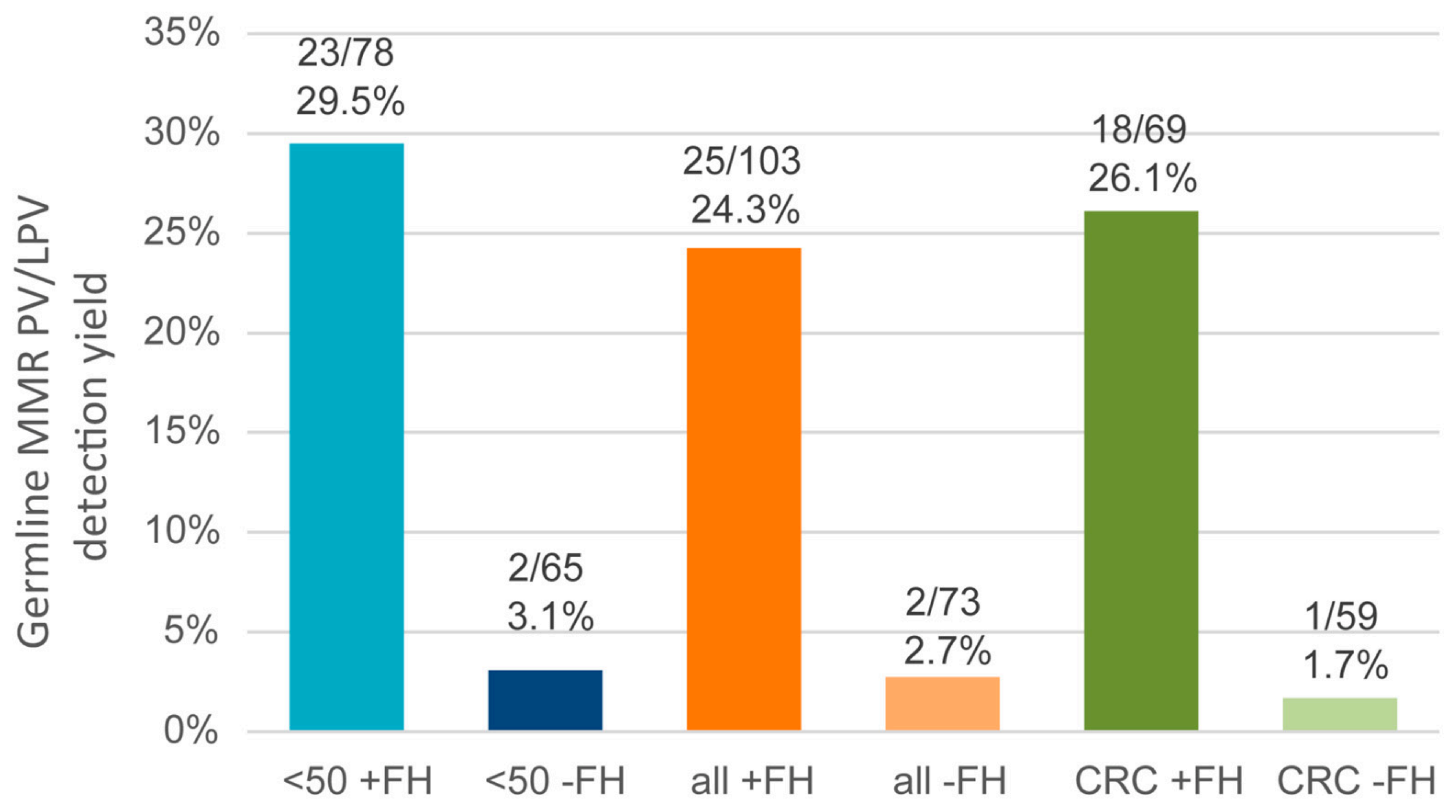
Detection yield of germline MMR PV/LPVs according to age at diagnosis and family history (FH) of LS-associated cancers. The highest detection yield of germline MMR PV/LPVs was observed among individuals diagnosed before the age of 50 years with a positive family history (<50 +FH), followed by CRC patients with a positive family history (CRC +FH) and all individuals with a positive family history (all +FH). Abbreviations: family history (FH), colorectal cancer (CRC), mismatch repair (MMR).

The majority of PV/LPVs in MMR genes (19/27) were detected among 128 patients with CRC (14.8%), while four were found in patients with EC (17.4%) (Table 1). Among CRC patients, the highest proportion of germline MMR PV/LPVs was observed in individuals with synchronous or metachronous CRC (Group 1b, 3/5, 60.0%), followed by patients in Group 1c (16/37, 43.2%) and Group 1d (11/29, 37.9%). Although the number of patients with multiple CRCs was limited, the high mutation yield observed in this subgroup, together with the elevated frequencies observed in family history–positive groups, supports the value of these clinical features in identifying individuals at increased likelihood of carrying germline MMR variants. As the clinical criteria used for group assignment were not mutually exclusive, some patients fulfilled criteria for both Group 1c and Group 1d. Therefore, mutation yields presented for these groups should be interpreted separately and not summed. In Group 1a, which included patients with CRC diagnosed before age 50 without FH, one germline MMR gene variant was detected. Importantly, CRC patients with a documented FH of LS-associated tumors had a significantly higher frequency of MMR PV/LPVs compared with those without FH (18/69, 26.1% vs. 1/59, 1.7%; Fisher’s exact test, OR = 20.47, 95% CI: 3.268–217.6, *p* = 0.0001) (Figure 2). The same association remained significant when the entire tested cohort was analyzed, including individuals with (25/103, 24.3%) and without FH (2/73, 2.7%) (Fisher’s exact test, OR = 11.38, 95% CI: 2.887–49.72, *p* = 0.0001) (Figure 2). These findings suggest that the presence of FH itself, rather than its specific configuration, is an important predictive factor for detecting germline MMR variants.

**Figure 3:**
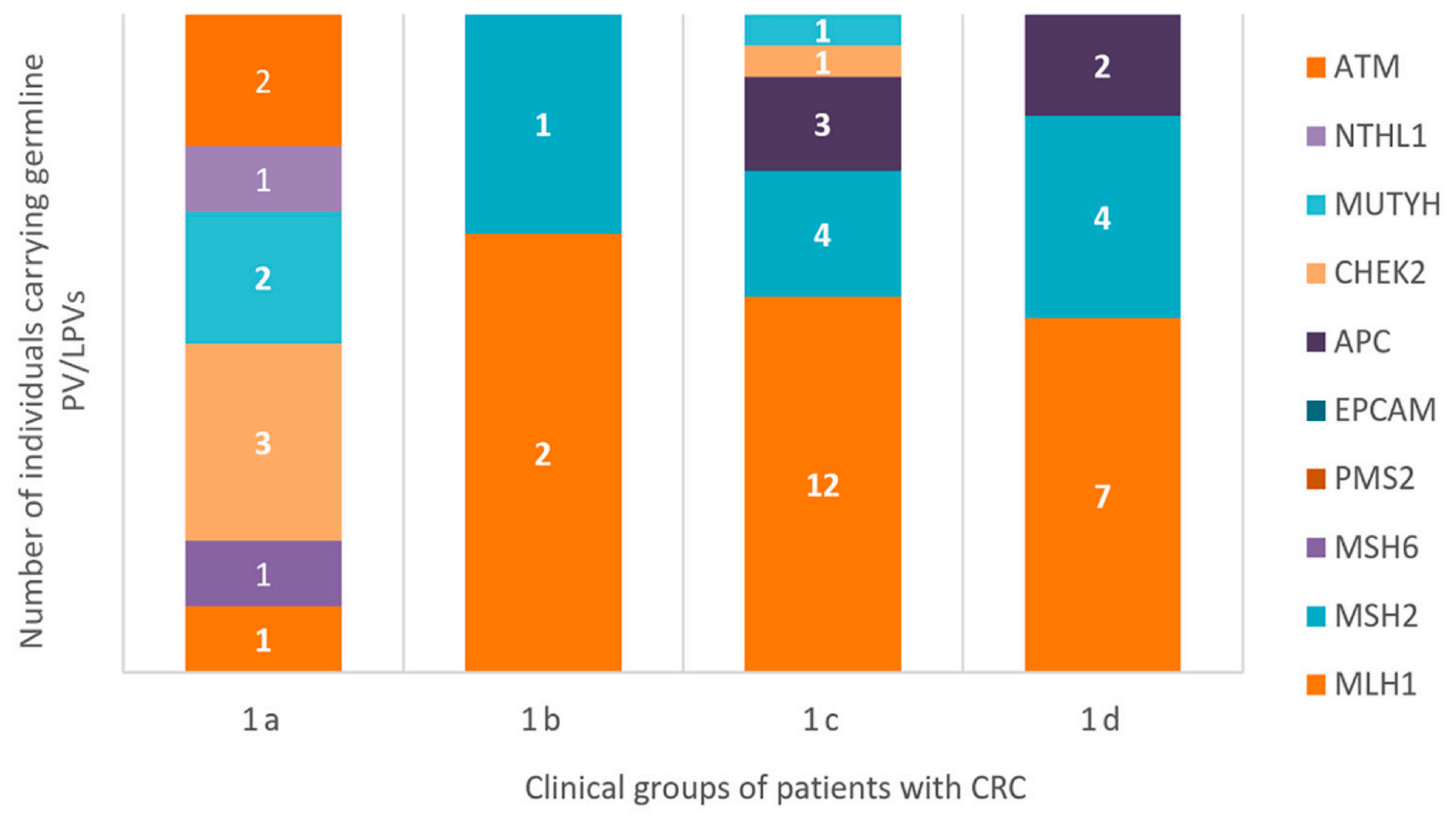
Distribution of individuals carrying germline PV/LPVs in MMR and non-MMR genes across CRC clinical subgroups (1a–1d). The figure shows the number of individuals identified with germline PV/LPVs in each gene across CRC subgroups. Counts represent individuals rather than the total number of detected variants; some individuals carried more than one PV/LPV. Groups 1c and 1d are partially overlapping, as some patients fulfilled criteria for both groups. *MLH1* variants were the most frequently detected, particularly in subgroups 1c and 1d, followed by *MSH2* and *APC* variants. Abbreviations: pathogenic variant (PV), likely pathogenic variant (LPV), colorectal cancer (CRC).

The distribution of individuals carrying germline PV/LPVs in MMR and non-MMR genes across CRC clinical subgroups (Groups 1a–1d) is shown in Figure 3.

In contrast to CRC, the number of patients with EC was limited, precluding meaningful comparisons between the clinical subgroups. Although higher mutation yields were observed in Groups 2b (2/4, 50.0%) and 2c (2/6, 33.3%) than in Group 2a (0/9), these findings should be interpreted with caution because of the small sample size. Among the 10 patients in Group 3 with multiple primary LS tumors, three with early-onset primary tumors had an MMR PV (30%). One *MLH1* PV/LPV was identified in Group 4, whereas no MMR PV/LPVs were identified in Group 5.

All detected MMR PV/LPV were found in individuals with an increased risk of being mutation carriers, as calculated by the MMRpro, PREMM model, and MMRPredict. Twenty-one individuals had an MMRpro-calculated carrier probability exceeding 50%, while the lowest probability among carriers was 21%. These observations are consistent with the use of prediction models as complementary tools in the clinical assessment of individuals referred for genetic counseling, although their diagnostic performance was not formally evaluated in this study.

### 2.3. Pathogenic/Likely Pathogenic Variants in Non-MMR Genes Associated with CRC

Among all tested individuals suspected of carrying germline MMR gene alterations, 16 non-MMR PV/LPV findings in genes associated or potentially associated with CRC were detected. These findings included 3 *APC*, 5 *CHEK2*, 4 *MUTYH*, 1 *NTHL1*, and 3 *ATM* PV/LPV findings (Table 2). Detailed characteristics of the individuals carrying these findings are described below.

Three patients were found to have PV/LPVs in the *APC* gene, including two PVs and one low-penetrance missense LPV, c.3920T>A (p.Ile1307Lys), with a moderate risk for CRC, particularly among the Ashkenazi Jewish population. Pathogenic *APC* variants were detected in very young patients with CRC, diagnosed before the age of 30, while one of them was also found to harbor a pathogenic *PALB2* variant.

All five *CHEK2* carriers (2.8%) had the same germline LPV, specifically the c.470T>C (p.Ile157Thr) missense mutation. Four of these individuals showed slight increases in the probabilities of carrying hereditary CRC syndrome according to MMRpro, PREMM, and MMRPredict models. Moreover, the fifth individual had a 72% probability of being an MMR mutation carrier and was concurrently found to have a heterozygous PV in the *MUTYH* gene.

Four pathogenic *MUTYH* variant findings were identified in three patients: one patient was confirmed by parental testing to carry two pathogenic *MUTYH* variants in trans, consistent with *MUTYH*-associated polyposis, whereas the remaining two patients were monoallelic carriers.

In three individuals, two PVs and one LPV were detected in the *ATM* gene. The person with an LPV was diagnosed with breast cancer and demonstrated an increased probability of testing positive for LS but not hereditary breast and ovarian cancer (HBOC) based on FH. The remaining two young patients with CRC without strongly positive FH carried the same heterozygous frameshift variant, c.1564_1565del (p.Glu522IlefsTer43).

A heterozygous pathogenic *NTHL1* variant was detected in a young female patient with early-onset CRC and no documented family history of LS-associated malignancies.

Additional heterozygous PV/LPVs were identified in *RAD51D*, *NBN*, *RAD50*, and *FANCM*. These genes are not currently established as major hereditary CRC predisposition genes, and some of these findings occurred concurrently with PV/LPVs in established cancer susceptibility genes.

### 2.4. Frequency of Variants of Uncertain Significance (VUS)

Variants of uncertain significance (VUS) in MMR genes were detected in eight patients (4.5%). These included three patients with *MSH2* VUS, of whom two patients carried the same heterozygous variant c.1597C>G (p.Leu533Val). In one patient, it was found alongside an LPV in the *MSH2* gene, while in the other patient, it coexisted with an LPV in the *FANCM* gene. One VUS was found in *MLH1*, 2 in *MSH6*, 1 in *PMS2*, and 1 in *EPCAM*. Additionally, 28 non-MMR VUS findings (15.9%) were detected, with the most frequently observed VUS in the *GALNT12, APC*, and *ATM* genes (Table 2).

## 3. Discussion

CRC represents a major public health burden in Serbia, while endometrial cancer also contributes substantially to cancer morbidity among women [13]. Against this background, the introduction of systematic genetic testing for LS at the IORS in 2018 represented an important milestone toward the structured identification of hereditary cancer predisposition and the future development of a nationwide screening program. Previous reports from Serbia focused primarily on individual cases and the functional characterization of specific MMR variants [18], highlighting the importance of accurate variant classification in LS. Building on these findings, our study expands the current knowledge by providing the first cohort-level analysis of LS-associated germline variants, thereby bridging the gap between case-based genetic observations and clinical implementation in hereditary CRC care.

This study provides the first comprehensive characterization of germline variants associated with LS in a clinically selected high-risk Serbian cohort. The overall frequency of MMR PV/LPVs (15.3%) among the 176 tested individuals represents a substantial diagnostic yield in this cohort selected according to clinical and familial criteria. Most PV/LPVs were identified in the *MLH1* gene (10.2%), followed by *MSH2* (4%) and *MSH6* (1.1%), a distribution that aligns with global trends showing that *MLH1* and *MSH2* PV/LPVs are more prevalent in LS than *MSH6* and *PMS2* PV/LPVs [19]. The predominance of *MLH1* PV/LPVs also aligns with reports from other European populations [1,20,21]. No pathogenic or likely pathogenic single nucleotide variants or small insertions/deletions were identified in *PMS2* or *EPCAM*, which may reflect cohort size, selection criteria, cohort-specific variation or potential technical challenges. However, the absence of such findings does not exclude exon-level deletions/duplications, large rearrangements, *EPCAM* 3′ deletions, or variants in technically challenging *PMS2* regions affected by *PMS2CL* homology. In short-read NGS data, this homology may lead to ambiguous read mapping and may limit the reliable detection or interpretation of variants in these regions. Larger studies and additional analyses will be necessary to determine whether this represents a broader regional pattern or simply reflects the characteristics of the present referral cohort.

One of the key findings was the high detection rate among individuals with a positive family history. This observation confirms the ongoing clinical value of detailed FH assessment and genetic counseling, even in the era of universal tumor screening. Although universal tumor screening is increasingly recommended, family history remains a powerful and cost-effective triage tool in resource-limited settings.

We identified two previously unreported *MLH1* variants, c.496_497insCACTT (p.Leu166SerfsTer3) and c.1759_1767delATGCTTGCC (p.Met587_Ala589del), both in young patients with LS-associated malignancies, including CRC. Their identification expands the known mutational spectrum of *MLH1* in the Serbian population. The two variants differ in their predicted molecular consequences: the frameshift variant is consistent with the established loss-of-function mechanism of *MLH1*-associated LS, whereas interpretation of the in-frame deletion relies on its localization within a mutational hotspot, its effect on protein length, and its absence from population databases. According to the applied ACMG/AMP framework, the frameshift variant was classified as pathogenic, whereas the in-frame deletion was classified as likely pathogenic. The absence of variant-specific functional, RNA, and informative segregation data remains a limitation. Further family-based and functional studies are needed to confirm their molecular effects and clinical relevance. In addition, the recurrent *MLH1* variants c.392C>G (p.Ser131Ter) and c.1275dupG (p.Gln426AlafsTer4) were identified in multiple unrelated individuals, raising the possibility of a founder effect or regional enrichment. The nonsense c.392C>G (p.Ser131Ter) *MLH1* variant has also been reported in unrelated individuals from the Macedonian population [22]. The recurrence of c.392C>G (p.Ser131Ter) in unrelated Serbian and Macedonian families may suggest a regionally enriched variant within Slavic or Balkan populations. However, haplotype analysis and population-based studies are required before any conclusions regarding founder effects or regional enrichment can be drawn.

Beyond MMR genes, PVs were also identified in non-MMR CRC-associated genes, including *CHEK2*, *APC*, *MUTYH*, *NTHL1*, and *ATM*. This finding is in line with other studies that report the co-occurrence of MMR PV/LPVs with PV/LPVs in non-MMR genes in hereditary CRC syndromes [23,24,25]. The detection of these variants highlights the limitations of an MMR-restricted testing strategy and supports the implementation of multigene panel testing in patients with suspected hereditary CRC syndromes. Emerging evidence from several studies suggests that genes such as *ATM* may confer a moderate CRC risk [26,27,28], while the National Comprehensive Cancer Network (NCCN) guidelines guidelines estimate the lifetime CRC risk for individuals with *ATM* pathogenic variants to be approximately 5–10%, compared to about 4.1% in the general population. However, current NCCN guidelines do not provide specific CRC screening recommendations for *ATM* carriers [29]. These findings emphasize the need for careful interpretation and individualized risk assessment. This study represents the first identification of an individual with confirmed *MUTYH*-associated polyposis (MAP) in our cohort, highlighting the importance of including *MUTYH* in multigene testing panels for patients with suspected hereditary CRC syndromes. Identification of monoallelic *MUTYH* carriers is also clinically relevant, as genetic counseling may provide important information regarding reproductive implications, including the possibility of biallelic pathogenic variant status in offspring if the reproductive partner is also a carrier. The monoallelic *NTHL1* finding should not be considered a molecular explanation for the patient’s CRC phenotype, as *NTHL1*-associated tumor syndrome is inherited in an autosomal recessive manner. Similarly, the *CHEK2* c.470T>C (p.Ile157Thr) variant represents a low-penetrance allele whose clinical relevance for CRC risk in isolation remains limited. Findings in *FANCM*, *NBN*, *RAD50*, and *RAD51D* should likewise be interpreted according to gene-specific evidence and should not be regarded as confirmed explanations for hereditary CRC predisposition.

VUS remain a challenge in clinical practice. In our cohort, VUS were identified in 4.5% of patients in MMR genes and in 15.9% in non-MMR CRC-associated genes, most frequently in *GALNT12, APC,* and *ATM*. Notably, the c.-18G>A variation in the *GALNT12* gene was found in a patient with a highly positive FH and MMRpro-calculated probability of being an MMR mutation carrier of 81%. This finding warrants further investigation, as *GALNT12* has previously been classified as a gene with moderate penetrance for CRC [30,31,32]. Patients with VUS, particularly those in high-risk genes and with a positive family history, should receive appropriate genetic counseling, and variant interpretation should be periodically reassessed as new evidence becomes available. Clinical management and surveillance should continue to be guided by the patient’s personal and family history rather than by the presence of a VUS itself.

This study has several limitations. First, the cohort represents a clinically selected high-risk population rather than a population-based sample. Accordingly, the observed distribution of MMR variants should be interpreted within the context of a clinically referred high-risk cohort and not as an estimate of the distribution of LS variants in the general Serbian population. Second, although next-generation sequencing was performed using a comprehensive panel, additional structural variants or regulatory region mutations may not have been fully captured. Third, the sample size limits the ability to perform robust genotype–phenotype correlation analyses or to draw firm conclusions regarding mutation patterns beyond this clinically selected cohort. Given the rarity of LS, assembling large, well-characterized national cohorts remains challenging even in larger healthcare systems. In recent years, our genetic counseling department for hereditary cancers has initiated efforts to increase awareness of hereditary CRC by organizing educational lectures for both clinicians and patients.

A further limitation concerns the inconsistent availability of tumor MMR status, reflecting variable implementation of MMR IHC and microsatellite instability (MSI) testing across institutions in Serbia. Current guidelines recommend universal tumor screening for LS in all newly diagnosed colorectal cancers using MMR IHC and/or MSI testing, together with assessment of personal and family cancer history [29]. In tumors showing MLH1/PMS2 loss, *BRAF* V600E testing and/or *MLH1* promoter methylation analysis is recommended to identify likely sporadic tumors and guide further germline evaluation [29].

Among the 27 patients with MMR-deficient tumors in our cohort, 13 (48.1%) were confirmed carriers of germline pathogenic or likely pathogenic variants in MMR genes (Table 2). Importantly, pMMR tumor status was not used as an exclusion criterion for germline testing, as LS-associated tumors may occasionally occur without detectable MMR deficiency [33]. In other Slavic countries, diagnostic algorithms for LS generally integrate MMR IHC, MSI testing, clinical criteria, and family history assessment to guide germline analysis [8,9,10,11,12,21]. Although specific approaches vary, many centers apply structured, stepwise molecular strategies aligned with international recommendations to optimize the identification of patients eligible for comprehensive genetic evaluation.

In Serbia, implementation of this sequential diagnostic pathway remains incomplete. Patients may undergo germline testing independently of tumor MMR or MSI findings, while *BRAF* testing is not systematically incorporated into the diagnostic pathway and *MLH1* promoter methylation analysis is currently unavailable in routine clinical practice. This limitation is particularly relevant in patients with MLH1/PMS2-deficient tumors in whom no germline MMR PV/LPV is identified, because sporadic MLH1-deficient tumors cannot be reliably distinguished from cases requiring further investigation for LS, constitutional *MLH1* epimutation, or other mechanisms of MMR deficiency.

The lack of an integrated tumor-first pathway may result in unnecessary germline testing in some patients and may complicate the interpretation of negative germline findings in others. Nevertheless, our results reflect current real-world practice and identify a specific gap in LS diagnostics in Serbia. Establishing a coordinated national algorithm that integrates MMR IHC and/or MSI testing, reflex *BRAF* V600E analysis and *MLH1* promoter methylation testing where appropriate, followed by appropriately selected germline testing, would improve patient selection and diagnostic efficiency. Such an approach will require closer collaboration and systematic data exchange among pathologists, oncologists, clinical geneticists, and molecular diagnostic laboratories, as well as improved access to tumor-based molecular testing.

Our next steps needed to advance the implementation of organized LS testing at the national level will entail performing a cost-effectiveness analysis, including inflation-adjusted real-world key costs, incremental cost-effectiveness ratios (ICERs), willingness-to-pay thresholds, and outcomes (deaths prevented, cancer cases avoided annually) stratified by age and sex. Approaches based on population-based genomic screening, universal tumor-first screening, risk-based testing guided by family history, and sequential testing strategies will need to be compared in relation to national willingness-to-pay thresholds per quality-adjusted life-year (QALY), which is of special importance in countries with limited healthcare resources such as Serbia. Most recent reports from both high-income and resource-limited regions suggest that upfront germline testing without prior tumor analysis may be the most favorable and cost-effective option [34,35]. Close, functional collaboration of clinicians, researchers, individuals at risk, patients, and patient advocates with national healthcare body representatives and policymakers has proven essential in other countries and should be used as a model going forward. This approach will be incorporated into the development of updated national guidelines for the management of CRC in Serbia.

Despite the noted limitations, this study provides important baseline data for the development of structured national LS screening strategies. Our findings contribute valuable data to global variant databases, support the development of risk-adapted testing strategies, and highlight the need for standardized national protocols for LS screening and cascade testing. Improving access to tumor-based screening, expanding reimbursement for cascade testing of relatives, and integrating genetic counseling and follow-up of individuals carrying PV/LPVs more systematically into oncology care remain critical priorities. Broader implementation of standardized LS screening pathways, in accordance with international guidelines, will be essential for earlier identification of pathogenic variant carriers and more effective cancer prevention in Serbia.

## 4. Patients and Methods

### 4.1. Subjects’ Selection

From January 2018 to October 2025, 215 individuals with suspected hereditary CRC predisposition were referred for genetic counseling from across Serbia. During pre-test genetic counseling, a detailed pedigree was obtained, including comprehensive personal and family cancer histories. MMR mutation carrier probabilities were calculated using MMRpro [36] implemented in CaGENE version 5.2 (UT Southwestern Medical Center, Dallas, TX, USA), the PREMM model [37], and MMRPredict [38]. Individuals who met the Amsterdam criteria and/or Bethesda guidelines [39], as well as relevant NCCN criteria [29], provided written informed consent approved by the Ethics Committee of the Institute for Oncology and Radiology of Serbia (IORS) before germline testing. Additionally, patients with a previously identified dMMR tumor status based on IHC results were referred for germline genetic testing. Some individuals without a diagnosis of malignant disease were referred for genetic counseling because of diagnosed colorectal polyposis or a positive family history of CRC. Genetic testing for eligible individuals was funded by the Serbian Health Insurance Fund. Individuals were considered ineligible for LS testing if they had a confirmed polyposis syndrome, clinically diagnosed familial adenomatous polyposis (FAP), or a low predicted probability of LS based on the models described above. However, individuals with limited or atypical polyposis in whom the underlying hereditary syndrome had not been established remained eligible for multigene testing. After excluding individuals with other hereditary cancer syndromes, unclear patterns of inheritance in the family history, or a low predicted probability of carrying pathogenic variants, 180 individuals were considered eligible for testing. By October 2025, germline testing had been completed in 176 individuals, who comprised the final analyzed cohort. The study cohort was divided into 5 groups and 10 subgroups based on the personal and/or FH of cancers (Table 1). All subjects, irrespective of the results obtained, were called for post-test genetic counselling at IORS, where further details regarding their families and possible additional testing were discussed. Patients were subsequently referred back to their treating oncologists to discuss further management. (Figure 1).

### 4.2. NGS Assay

Genomic DNA was extracted from whole blood using the ABI Prism™ 6100 Nucleic Acid PrepStation and Blood-Prep Kit (Applied Biosystems, Foster City, CA, USA). Germline genetic testing was performed by next-generation sequencing (NGS) using the TruSight Hereditary Cancer Panel (catalogue no. 20024717; Illumina, San Diego, CA, USA), together with the Illumina DNA Prep with Enrichment Tagmentation kit (catalogue no. 20025524; Illumina, San Diego, CA, USA), according to the manufacturer’s instructions. Sequencing was performed on the Illumina MiSeq Sequencing System (Illumina, San Diego, CA, USA). The analysis was performed using the GRCh37/hg19 reference genome. Sequencing quality was assessed according to predefined criteria. Variants were considered for further interpretation when they fulfilled the following parameters: read depth ≥ 100×, variant allele fraction consistent with germline heterozygous or homozygous status, Phred base quality ≥ 30, mapping quality ≥ 30, absence of significant strand bias, and concordance with visual inspection in the Integrative Genomics Viewer (IGV). Samples not meeting the predefined quality criteria were excluded from analysis or resequenced when sufficient DNA was available.

The primary analysis focused on germline variants in the coding regions and consensus splice sites of LS-associated genes, including the MMR genes *MLH1*, *MSH2*, *MSH6* and *PMS2*, as well as *EPCAM*. In addition, further analysis included high- and moderate-penetrance CRC-associated genes (*APC*, *MUTYH*, *CHEK2*, *SMAD4*, *BMPR1A*, *STK11*, *TP53*, *PTEN*, *POLE*, *POLD1*), as well as genes with emerging evidence of CRC association (*ATM*, *NTHL1*, *GREM1*, *GALNT12*). Variants detected in additional genes included in the panel, which are not primarily associated with CRC risk, were also reported in this study when considered relevant.

### 4.3. Variant Annotation

Data analysis and base calling were performed using MiSeq Reporter software version 2.5.1 (Illumina, San Diego, CA, USA) [40]. Variant calling and annotation were conducted using Illumina Variant Studio software for variant call format (VCF) v4.1 files generated during secondary analysis. An in-house variant database was established using Geneticist Assistant software version 1.8.3 (SoftGenetics, State College, PA, USA) for variant annotation and internal variant tracking. The functional significance of detected variants was evaluated using public databases, relevant literature, and in silico prediction tools, as previously described [41]. Variant interpretation and classification were performed according to the ACMG/AMP guidelines [42]. All reportable pathogenic and likely pathogenic variants, as well as selected variants of uncertain significance considered relevant for the study, were manually inspected using IGV [41].

PV and LPV, corresponding to ACMG classes 4 and 5, and VUS, corresponding to ACMG class 3, are presented in Supplementary Table S1 and Supplementary Table S2, respectively. The reference transcripts used for variant annotation and nomenclature are listed together with the reported variants in these Supplementary Tables. Variants classified as likely benign or benign were considered common polymorphisms and were not included in the main analysis.

### 4.4. Limitations

The applied NGS workflow was primarily used for the detection of single nucleotide variants and small insertions/deletions within the targeted regions. Exon-level deletions/duplications, large genomic rearrangements, and *EPCAM* 3′ deletions were not systematically assessed. In addition, due to the high sequence homology between *PMS2* and the *PMS2CL* pseudogene, assessment of some *PMS2* regions may be technically limited using short-read panel-based sequencing. Therefore, the results for *PMS2* and *EPCAM* refer to sequence-level variants detectable by the applied panel-based NGS approach and should not be interpreted as complete exclusion of all possible pathogenic alterations in these genes.

### 4.5. MMR Immunohistochemistry Data Collection

MMR IHC results were collected retrospectively from the clinical and pathology documentation available at the time of patient referral for genetic counseling and germline genetic testing. MMR IHC was performed as part of routine diagnostic work-up at different pathology departments across Serbia and was not a prerequisite for study inclusion or germline testing.

MMR IHC findings were recorded according to the final interpretation provided in the original pathology reports.

Because the analyses were performed at multiple external institutions, detailed information regarding antibody clones, staining platforms, internal controls, and centralized slide review was not consistently available.

### 4.6. Statistics

Participant characteristics and sequencing outcomes were summarized using descriptive statistics. Categorical variables are presented as frequencies and percentages. Fisher’s exact test was used, where appropriate, to evaluate associations between categorical variables. Statistical analyses were performed using GraphPad Prism version 10. A two-sided *p*-value < 0.05 was considered statistically significant.

## 5. Conclusions

This study provides the first systematic characterization of LS-associated germline variants in a high-risk Serbian cohort, providing an important foundation for hereditary CRC research in the region.

The observed variant spectrum, characterized by a predominance of *MLH1* variants and the absence of detectable pathogenic or likely pathogenic sequence-level variants in *PMS2* and *EPCAM*, reflects the variant spectrum identified using the applied panel-based NGS approach in this clinically selected cohort.

The identification of previously unreported and recurrent *MLH1* variants expands current knowledge of LS genetic architecture in Southeastern Europe and may warrant further investigation of possible regional enrichment. In addition, the detection of pathogenic variants in non-MMR genes supports the use of multigene panel testing in hereditary CRC diagnostics.

Limited awareness and incomplete implementation of LS screening strategies in Serbia continue to represent significant barriers to early detection and cascade testing. These findings underscore the need for standardized national screening protocols, improved access to genetic testing, and systematic identification and follow-up of at-risk individuals.

Overall, this study contributes valuable data to global variant databases and provides a foundation for future population-based, functional, and health-economic studies aimed at improving precision prevention strategies in hereditary CRC.

## Figures and Tables

**Table 1:** Distribution of study participants according to clinical criteria for LS testing and frequency of pathogenic/likely pathogenic (PV/LPV) mismatch repair (MMR) variants detected in each subgroup. Abbreviations: colorectal cancer (CRC), endometrial cancer (EC), family history (FH), first-degree relative (FDR), second-degree relative (SDR), Lynch syndrome (LS), ovarian cancer (OC), mismatch repair (MMR), pathogenic/likely pathogenic variant (PV/LPV), calculated probability of LS (CP).

Characteristics of Tested Groups	No.	% (n = 176)	No. of MMR Mutated	% *
**GROUP 1**				
**Patients with CRC**	128	72.7	19	14.8
*Group 1a* Patients diagnosed before the age of 50 with no FH	53	30.1	1	1.9
*Group 1b* Patients diagnosed at any age with synchronous and/or metachronous CRC	5	2.8	3	60
*Group 1c* ^†^ Patients diagnosed at any age with FH of at least one FDR/SDR with CRC or another LS tumour before the age of 50	37	21	16	43.2
*Group 1d* ^†^ Patients diagnosed at any age with FH of at least two first or second-degree relatives with CRC or another LS tumour at any age	29	16.5	11	37.9
**GROUP 2**				
**Patients with EC**	23	13.1	4	17.4
*Group 2a* Patients diagnosed before the age of 50 with no FH	9	5.1	0	0
*Group 2b* ^†^ Patients diagnosed at any age with FH of at least one FDR/SDR with another LS tumour before the age of 50	4	2.3	2	50
*Group 2c* ^†^ Patients diagnosed at any age with FH of at least two FDR/SDRs with another LS tumour at any age	6	3.4	2	33.3
**GROUP 3**				
Patients diagnosed at any age with multiple primary LS tumours (EC, CRC, OC, duodenal, pancreatic)	10	5.7	3	30
**GROUP 4**				
Patients with non-typical LS cancer (breast, Fallopian tube cancer, pancreatic) with FH of FDR/SDR with any type of LS tumour including ≥1 diagnosed <50 and/or with CP higher than 5% for LS	10	5.7	1	10
**GROUP 5**				
Healthy persons or persons with benign dysplasia with FH of FDR/SDR with any type of LS tumour including ≥1 diagnosed <50 and/or with CP higher than 5% for LS	5	2.8	0	0

* Percentage of individuals carrying a PV/LPV MMR variant within the respective subgroup. ^†^ Some individuals fulfilled criteria for both Groups 1c and 1d; therefore, these subgroups are not mutually exclusive.

**Table 2:** Distribution of detected germline variant findings in the study cohort (n = 176). PV/LPVs (classes 4 and 5) were predominantly detected in MMR genes, particularly in *MLH1* and *MSH2*, while variants of uncertain significance (VUS) (class 3) were more frequently observed in non-MMR genes. Abbreviations: pathogenic/likely pathogenic (PV/LPV), mismatch repair (MMR), variants of uncertain significance (VUS).

	Classes 4 and 5 (PV/LPV)		Class 3 (VUS)	
	n	%	n	%
**MMR gene and *EPCAM* variants detected**	27	15.3	8	4.5
*MLH1*	18	10.2	1	0.6
*MSH2*	7	4	3	1.7
*MSH6*	2	1.1	2	1.1
*PMS2*	0	0.0	1	0.6
*EPCAM*	0	0.0	1	0.6
**Non-MMR gene variants detected**	16	9.1	28	15.9
*APC*	3	1.7	5	2.8
*BMPR1A*	0	0.0	0	0.0
*CHEK2*	5	2.8	2	1.1
*MUTYH*	4	2.3	2	1.1
*SMAD4*	0	0.0	0	0.0
*STK11*	0	0.0	0	0.0
*TP53*	0	0.0	2	1.1
*PTEN*	0	0.0	2	1.1
*POLE*	0	0.0	1	0.6
*POLD1*	0	0.0	3	1.7
*NTHL1*	1	0.6	1	0.6
*GREM1*	0	0.0	1	0.6
*GALNT12*	0	0.0	4	2.3
*ATM*	3	1.7	5	2.8

**Table 3:** Correlation between MMR immunohistochemistry (IHC) patterns, tumor types, and germline genetic findings. Abbreviations: immunohistochemistry (IHC), colorectal cancer (CRC), endometrial cancer (EC), mismatch repair (MMR), pathogenic/likely pathogenic variant (PV/LPV), variant of uncertain significance (VUS), Lynch syndrome (LS).

MMR IHC Pattern	No. of Patients	Type of Tumor	Germline PV/LPV Detected	No. MMR PV/LPV Detected	VUS Detected
MLH1/PMS2 loss	11	CRC (n = 8); EC (n = 2); Pancreatic Ca (n = 1)	*MLH1* PV/LPV (n = 7)	4	0
MSH2/MSH6 loss	6	CRC (n = 5); EC (n = 1)	*MSH2* PV/LPV (n = 4)	2	1 *
Isolated MSH2 loss	4	CRC	*MSH2* PV/LPV (n = 1)	3	0
Isolated MSH6 loss	2	CRC	*MSH6* PV/LPV (n = 0)	2	0
Isolated PMS2 loss	2	CRC	*PMS2* PV/LPV (n = 0)	2	0
Other/complex patterns (≥3 proteins lost)	2	CRC (n = 1); EC (n = 1)	MMR PV/LPV (n = 1, *MLH1*)	1	0
pMMR (retained expression)	13	CRC (n = 12); multiple LS tumor (n = 1)	MMR PV/LPV (n = 0)	13	1 *
Total	40	/	13	27	2

* VUS was detected in one patient who also carried an MMR PV/LPV, and in one patient without an MMR PV/LPV; both patients are already included in the respective groups and do not represent additional cases.

## Data Availability

De-identified aggregate data and variant-level information generated and analyzed in this study are provided within the manuscript and Supplementary Materials. Additional individual-level data are not publicly available due to ethical restrictions and patient privacy considerations, as they may contain information that could compromise participant confidentiality.

## Supplementary Materials

The following supporting information can be downloaded at https://www.mdpi.com/article/10.3390/ijms27156652/s1.

## Abbreviations

The following abbreviations are used in this manuscript:

ACMG	American College of Medical Genetics and Genomics
AMP	Association for Molecular Pathology
CP	Predicted probability of Lynch syndrome calculated using validated risk prediction models
CRC	Colorectal cancer
EC	Endometrial cancer
EHTG	European Hereditary Tumour Group
ESCP	European Society of Coloproctology
FAP	Familial adenomatous polyposis
FDR	First-degree relative
FH	Family history
HNPCC	Hereditary nonpolyposis colorectal cancer
IGV	Integrative Genomics Viewer
IORS	Institute for Oncology and Radiology of Serbia
LPV	Likely pathogenic variants
LS	Lynch syndrome
MMR	Mismatch repair
MSI	Microsatellite Instability
NCCN	National Comprehensive Cancer Network
NGS	Next-generation sequencing
OC	Ovarian cancer
Pts	Patients
PV	Pathogenic variants
SDR	Second-degree relative
VUS	Variant/s of uncertain significance
ICER	Incremental Cost-Effectiveness Ratio
QALY	Quality-Adjusted Life Year
