# Supplementary material for: Genetic Landscape of Lynch Syndrome in a High-Risk Serbian Cohort: Predominance of MLH1 Variants and Implications for Risk-Based Testing
Source: Int J Mol Sci. 2026 Jul 25;27(15):6652. doi: 10.3390/ijms27156652 (PMC13466496; doi:10.3390/ijms27156652)
Supplement: Supplementary file 1 [file ijms-27-06652-s001.zip › ijms-4417280-supplementary.pdf]

Supplementary Materials: Genetic Landscape of Lynch Syndrome in a High-Risk Serbian Cohort: Predominance of *MLH1* Variants and Implications for Risk-Based Testing

Marija Djordjic Crnogorac*, Valentina Karadzic, Teodora Cato, Milena Cavic, Neda Nikolic, Jelena Spasic, Fedja Djordjevic, Vladimir Jokic, Milan Kocic, Miroslav Djurasinovic, Biljana Kukic, Srdjan Nikolic, Marija Ristic, Marijana Milovic, Ana Krivokuca*

Supplementary Table S1. Pathogenic and likely pathogenic germline variants identified in the study cohort.

Supplementary Table S2. Germline variants of uncertain significance identified in the study cohort.

Supplementary Table S3. Individual clinical, immunohistochemical, and germline genetic findings in patients with available MMR immunohistochemistry (IHC) results.

Supplementary Table S4. Classification framework and supporting evidence for the two previously unreported *MLH1* variants identified in the study cohort.

Supplementary Table S1. Pathogenic and likely pathogenic germline variants identified in the study cohort.

Gene	Variant	No. of individual s	Exon/Intron position	Class of variant
MLH1 (NM_000249.3)	c.2252_2253delAA (p.Lys751SerfsTer3)	1	19	5
	c.392C>G (p.Ser131Ter)	3	5	5
	c.86C>G (p.Ala29Gly)	1	1	5
	c.496_497insCACTT (p.Leu166SerfsTer3)	2	6	5
	c.1759_1767delATGCTTGCC (p.Met587_Ala589del)	1	16	4
	c.1275dupG (p.Gln426AlafsTer4)	4	12	5
	c.244A>G (p.Thr82Ala)	1	3	5
	c.116+1G>A	1	1	5
	c.793C>T (p.Arg265Cys)	1	3	5
	c.350C>T (p.Thr117Met)	1	4	5
	c.790+1G>A	1	9	5
	c.199G>A (p.Gly67Arg)	1	2	5
MSH2 (NM_000251.3)	c.2634+1G>C	1	15	4
	c.1661G>A (p.Ser554Asn)	1	3	4
	c.942+3A>T	1	5	5
	c.1705_1706del (p.Glu569fs)	1	11	5
	c.2131C>T (p.Arg711Ter)	1	13	5
	c.2269dup (p.Tyr757fs)	1	14	5
	c.1225C>T (p.Gln409Ter)	1	7	5
MSH6 (NM_000179.3)	c.3699_3702del (p.Lys1233AsnfsTer6)	1	8	5
	c.3514dup (p.Arg1172fs)	1	6	5
APC (NM_000038.6)	c.3306C>G (p.Tyr1102Ter)	1	16	5
	c.3920T>A (p.Ile1307Lys)	1	16	4
	c.2626C>T (p.Arg876Ter)	1	16	5
CHEK2 (NM_007194.4)	c.470T>C (p.Ile157Thr)	5	4	4
	c.1103G>A (p.Gly368Asp)	1	13	5

MUTYH *(NM_001048174.2)*	c.650G>A (p.Arg217His)	2	8	5
	c.1353_1355delGGA (p.Glu452del)	1	14	5
NTHL1 *(NM_002528.7)*	c.244C>T (p.Gln82Ter)	1	2	5
ATM *(NM_000051.4)*	c.1564_1565delGA (p.Glu522IlefsTer43)	2	10	5
	c.8578_8580delTCT (p.Ser2860del)	1	58	4

Supplementary Table S2. Germline variants of uncertain significance identified in the study cohort.

Gene	Variant	No. of individuals	Exon/Intron position
MLH1 *(NM_000249.3)*	c.1270G>A (p.Ala424Thr)	1	12
MSH2 *(NM_000251.3)*	c.1597C>G (p.Leu533Val)	2	10
	c.260C>T (p.Ser87Phe)	1	2
MSH6 *(NM_000179.3)*	c.2864T>C (p.Leu955Pro)	1	4
	c.3104G>T (p.Arg1035Leu)	1	4
PMS2 *(NM_000535.7)*	c.1567T>A (p.Ser523Thr)	1	11
EPCAM *(NM_002354.3)*	c.184+14C>G	1	2
APC *(NM_000038.6)*	c.531T>C (p.Asn177=)	1	5
	c.4073C>T (p.Ala1358Val)	1	16
	c.5110A>G (p.Lys1704Glu)	1	16
	c.5528C>T (p.Pro1843Leu)	1	16
	c.6095T>C (p.Ile2032Thr)	1	16
CHEK2 *(NM_007194.4)*	c.482A>G (p.Glu161Gly)	1	4
	c.1067C>T (p.Ser356Leu)	1	10
MUTYH *(NM_001048174.2)*	c.2T>C (p.Met1Thr)	1	1
	c.46G>T (p.Ala16Ser)	1	2
TP53 *(NM_000546.6)*	c.37C>T (p.Pro13Ser)	1	2
	c.993+303T>A	1	9
PTEN *(NM_000314.8)*	c.-475G>A	1	5′ UTR
	c.-466G>T	1	5′ UTR
POLE *(NM_006231.4)*	c.2865-3C>T	1	24
	c.3179G>A (p.Arg1060His)	1	26

POLD1 (NM_002691.4)	c.1930G>A (p.Asp644Asn)	1	16
	c.2429C>T (p.Ala810Val)	1	20
NTHL1 (NM_002528.7)	c.503T>C (p.Ile168Thr)	1	3
GREM1 (NM_013372.7)	c.10A>G (p.Thr4Ala)	1	2
GALNT12 (NM_024642.5)	c.-18G>A	1	5′ UTR
	c.-1C>A	1	5′ UTR
	c.19C>T (p.Arg7Trp)	1	1
	c.136G>C (p.Gly46Arg)	1	1
ATM (NM_000051.4)	c.7740_7742delAAG (p.Arg2580del)	1	52
	c.4165A>G (p.Thr1389Ala)	1	28
	c.6497T>C (p.Val2166Ala)	1	45
	c.6808C>T (p.Leu2270Phe)	1	47
	c.7198A>G (p.Arg2400Gly)	1	49

Supplementary Table S3. Individual clinical, immunohistochemical, and germline genetic findings in patients with available MMR immunohistochemistry (IHC) results. Abbreviations: immunohistochemistry (IHC), colorectal cancer (CRC), endometrial cancer (EC), mismatch repair (MMR), pathogenic/likely pathogenic variant (PV/LPV), variant of unknown significance (VUS), Lynch syndrome (LS), wild type (WT, no germline MMR PV/LPV detected)

ID	Tumor type	MMR IHC pattern	Germline gene mutation	Variant	Classification
P01	CRC	MLH1/PMS2 loss	WT	-	-
P02	CRC	Isolated MSH2 loss	WT	-	-
P03	CRC	pMMR	WT	-	-
P04	CRC	Isolated MSH6 loss	WT	-	-
P05	CRC	MLH1/PMS2 loss	*MLH1*	NM_000249.3(MLH1):c.392C>G (p.Ser131Ter)	Pathogenic
P06	CRC	MSH2/MSH6 loss	*MSH2*	NM_000251.3(MSH2):c.1705_1706del (p.Glu569fs)	Pathogenic
P07	CRC	MLH1/PMS2 loss	*MLH1*	NM_000249.3(MLH1):c.86C>G (p.Ala29Gly)	Pathogenic
P08	CRC	All MMR proteins loss	*MLH1*	NM_000249.3(MLH1):c.392C>G (p.Ser131Ter)	Pathogenic
P09	CRC	MSH2/MSH6 loss	WT	-	-
P10	CRC	MLH1/PMS2 loss	*MLH1*	NM_000249.3(MLH1):c.1275dupG (p.Gln426AlafsTer4)	Pathogenic
P11	CRC	MSH2/MSH6 loss	*MSH2*	NM_000251.3(MSH2):c.2634+1G>C	Likely pathogenic

P12	EC	MLH1/MSH6/PMS2 loss	WT	-	-
P13	CRC	MSH2/MSH6 loss	*MSH2*	NM_000251.3(MSH2):c.2269dup (p.Tyr757fs)	Pathogenic
P14	CRC	MSH2/MSH6 loss	*MSH2*	NM_000251.3(MSH2):c.1661G>A (p.Ser554Asn) NM_000251.3(MSH2):c.1597C>G (p.Leu533Val)	Likely pathogenic and VUS
P15	CRC	pMMR	WT	-	-
P16	CRC	pMMR	WT	-	-
P17	CRC	pMMR	WT	-	-
P18	CRC	MLH1/PMS2 loss	*MLH1*	NM_000249.3(MLH1):c.793C>T (p.Arg265Cys)	Pathogenic
P19	CRC	pMMR	WT	-	-
P20	CRC	Isolated MSH6 loss	WT	-	-
P21	CRC	pMMR	WT	-	-
P22	CRC	pMMR	WT	-	-
P23	CRC	pMMR	WT	-	-
P24	CRC	MLH1/PMS2 loss	WT	-	-
P25	CRC	Isolated PMS2 loss	WT	-	-
P26	CRC	pMMR	*MSH2*	NM_000251.3(MSH2):c.260C>T (p.Ser87Phe)	VUS
P27	CRC	pMMR	WT	-	-
P28	CRC	MLH1/PMS2 loss	*MLH1*	NM_000249.3(MLH1):c.1275dupG (p.Gln426AlafsTer4)	Pathogenic
P29	EC	MSH2/MSH6 loss	WT	-	-
P30	CRC	MLH1/PMS2 loss	*MLH1*	NM_000249.3(MLH1):c.790+1G>A	Pathogenic
P31	CRC	pMMR	WT	-	-
P32	Pancreatic Ca	MLH1/PMS2 loss	*MLH1*	NM_000249.3(MLH1):c.496_497insCACTT (p.Leu166SerfsTer3)	Pathogenic
P33	CRC	Isolated MSH2 loss	WT	-	-
P34	CRC	pMMR	WT	-	-
P35	CRC	Isolated PMS2 loss	WT	-	-
P36	CRC	Isolated MSH2 loss	*MSH2*	NM_000251.3(MSH2):c.1225C>T (p.Gln409Ter)	Pathogenic
P37	CRC	Isolated MSH2 loss	WT	-	-
P38	EC	MLH1/PMS2 loss	WT	-	-
P39	EC	MLH1/PMS2 loss	WT	-	-
P40	CRC	pMMR	WT	-	-

Supplementary Table S4. Classification framework and supporting evidence for the two previously unreported *MLH1* variants identified in the study cohort.

Gene	*MLH1*	*MLH1*
Reference transcript	NM_000249.3	NM_000249.3
HGVS cDNA nomenclature	c.496_497insCACTT	c.1759_1767delATGCTTGCC
HGVS protein nomenclature	p.Leu166SerfsTer3	p.Met587_Ala589del
Variant type	Frameshift insertion resulting in a premature termination codon	In-frame deletion of three amino acids
Functional domain	N-terminal ATPase domain	C-terminal PMS2-binding domain
Population databases	Absent from large population databases, including gnomAD	Absent from large population databases, including gnomAD
ClinVar/LOVD	Not reported at the time of analysis	Not reported at the time of analysis
Clinical findings	Detected in two unrelated young patients with tumors consistent with Lynch syndrome	Detected in a young patient with colorectal cancer
Tumor findings	Loss of MLH1/PMS2 expression by immunohistochemistry was documented in one available tumor	No informative IHC/MSI data available
Segregation data	No informative segregation data available	No informative segregation data available
Functional/RNA evidence	No variant-specific functional or RNA studies available	No variant-specific functional or RNA studies available
ACMG/AMP criteria and rationale	**PVS1:** Frameshift introducing a premature termination codon predicted to undergo nonsense-mediated decay; loss of function is an established disease mechanism.<br><br>**PM2_Supporting:** Absent from population databases.	**PM1_Strong:** In-frame deletion affecting a well-established mutational hotspot with limited benign variation.<br><br>**PM4:** In-frame deletion altering protein length.<br><br>**PM2_Supporting:** Absent from population databases.
Final classification	Pathogenic	Likely pathogenic
